# Community structure and association network of prokaryotic community in surface sediments from the Bering-Chukchi shelf and adjacent sea areas

**DOI:** 10.3389/fmicb.2023.1312419

**Published:** 2024-01-09

**Authors:** Changliang Xie, Hong Ouyang, Hu Zheng, Maoting Wang, Junning Gu, Zhaohui Wang, Yali Tang, Lijuan Xiao

**Affiliations:** College of Life Science and Technology, Jinan University, Guangzhou, China

**Keywords:** bacteria, Pacific Arctic, sediment, metabarcoding, 16S rRNA gene, association network

## Abstract

The Bering-Chukchi shelf is one of the world’s most productive areas and characterized by high benthic biomass. Sedimentary microbial communities play a crucial role in the remineralization of organic matter and associated biogeochemical cycles, reflecting both short-term changes in the environment and more consistent long-term environmental characteristics in a given habitat. In order to get a better understanding of the community structure of sediment-associated prokaryotes, surface sediments were collected from 26 stations in the Bering-Chukchi shelf and adjacent northern deep seas in this study. Prokaryote community structures were analyzed by metabarcoding of the 16S rRNA gene, and potential interactions among prokaryotic groups were analyzed by co-occurrence networks. Relationships between the prokaryote community and environmental factors were assessed. Gammaproteobacteria, Alphaproteobacteria, and Flavobacteriia were the dominant bacterial classes, contributing 35.0, 18.9, and 17.3% of the bacterial reads, respectively. The phototrophic cyanobacteria accounted for 2.7% of the DNA reads and occurred more abundantly in the Bering-Chukchi shelf. Prokaryotic community assemblages were different in the northern deep seas compared to the Bering-Chukchi shelf, represented by the lowered diversity and the increased abundant operational Taxonomic Units (OTU), suggesting that the abundant taxa may play more important roles in the northern deep seas. Correlation analysis showed that latitude, water depth, and nutrients were important factors affecting the prokaryote community structure. Abundant OTUs were distributed widely in the study area. The complex association networks indicated a stable microbial community structure in the study area. The high positive interactions (81.8–97.7%) in this study suggested that symbiotic and/or cooperative relationships accounted for a dominant proportion of the microbial networks. However, the dominant taxa were generally located at the edge of the co-occurrence networks rather than in the major modules. Most of the keystone OTUs were intermediately abundant OTUs with relative reads between 0.01 and 1%, suggesting that taxa with moderate biomass might have considerable impacts on the structure and function of the microbial community. This study enriched the understanding of prokaryotic community in surface sediments from the Bering-Chukchi shelf and adjacent sea areas.

## Introduction

Prokaryotes play a key role in decomposing organic matter and recycling nutrients in the ocean (Azam et al., 1983; Walker et al., 2023). Marine sediments are important habitats for prokaryotic microorganisms, and harbor diverse microbial communities and huge genetic variations (Zeng et al., 2011). Sediment prokaryotic communities are major mediators at the sediment-water interfaces and within marine sediments (Walker et al., 2023), and can reflect both short-term changes associated with rapid sea ice loss and warming and more consistent long-term environmental characteristics in a given habitat (Zinger et al., 2011; Fuhrman et al., 2015).

The Arctic Ocean is one of the unique regions on the planet with extremely low water temperature and seasonal darkness, and the microorganisms in this habitat are significantly different from those in tropical and temperate seas in terms of species, metabolic mechanism, and community structure (Deming, 1986). Biological surveys on the Arctic Ocean began in 1893 by the Nansen Expedition. Due to the limitation of observation techniques, the biological abundance and diversity of the Arctic Ocean were greatly underestimated in the early research period (Von Quillfeldt, 1997). During the last decade, technological progress in molecular ecology and environmental DNA sequencing has substantially boosted our understanding of marine microbes, greatly unveiling the diversity of microflora in the Arctic Ocean especially by means of high-throughput sequencing (HTS) technologies (Galand et al., 2010; Pedrós-Alió et al., 2015; Kurdy et al., 2023). Results suggested huge microbial diversity in the Pacific Arctic and Arctic Ocean, and Pseudomonadota mostly Gammaproteobacteria and Alphaproteobacteria are dominated in both water and sediment microbial communities similar to the worldwide marine environments (Pedrós-Alió et al., 2015; Dong et al., 2017; Rapp et al., 2018; Fang et al., 2019; Kurdy et al., 2023).

The Bering-Chukchi shelf covers an uninterrupted coastal shelf that spans the Pacific Arctic region, and is one of the world’s most bio-productive areas and characterized by high benthic biomass due to the persistent flow of nutrient-rich waters from the north Pacific through the Bering Strait (Hill et al., 2018; Huntington et al., 2020; Lalande et al., 2021). It has been the area with the largest reduction of sea ice and the strongest desalination in Arctic Ocean, and is regarded as a barometer of global change and amplifier of global warming (Previdi et al., 2021; Dai and Jenkins, 2023; Gao et al., 2023). These changes may influence the carbon sink from the upper water to the bottom sea, as well as the benthic community structure especially for the microbial communities (Rapp et al., 2018; O’Daly et al., 2020; Walker et al., 2023). However, benthic prokaryotic community structure has been limitedly reported in the Bering-Chukchi shelf and adjacent sea areas (Dong et al., 2017; Sun et al., 2022; Walker et al., 2023).

In order to get a better understanding of the community structure of sediment-associated prokaryotes, surface sediments were collected from 26 stations in the Bering-Chukchi shelf and adjacent northern deep seas in this study. Prokaryote community structures were analyzed by metabarcoding of the 16S rRNA gene, and potential interactions among prokaryotic groups were analyzed by the co-occurrence networks. Biogenic elements including total organic carbon (TOC), biogenic silica (BSi), total nitrogen (TN), and total phosphorus (TP), were measured in the sediment samples. Relationships between prokaryotic community and environmental factors were assessed. The purpose of this study is (1) to compare the benthic prokaryotic community structures between the Bering-Chukchi shelf and the northern deep seas, (2) to investigate environmental correlations with prokaryotic community, and (3) to assess potential interactions among prokaryotic groups.

## Materials and methods

### Study areas

The Bering-Chukchi shelf locates in the Pacific Arctic Ocean, and plays an important role in water exchange between the Arctic Ocean and the northern Pacific Ocean. The Bering Sea, with latitude from 51°N-66°N, is one of the “high-nutrient and low-chlorophyll” (HNLC) sea areas in the world (Lin et al., 2016). Sediments in the Bering Sea are mainly composed of terrigenous materials, and the nearshore sediments are constituted by coarse sand with gravels and shells and gradually replaced by fine clay with the increase of offshore distance (Chen, 2012). The Bering Strait is an important channel connecting the Bering Sea and the Chukchi Sea, and sediments near the Chukchi Sea are mainly composed by coarse-gravels and stone-pebbles (Yu, 2008). The Chukchi Sea, with latitude from 65°N to 75°N, is the most productive area in the Arctic shelf (Huntington et al., 2020; Lalande et al., 2021). Sediments in the Chukchi Sea shelf are composed of sand, silt, and clay, and become finer with the increase of latitude (Wang et al., 2015).

### Sample collection

**FIGURE 1:**
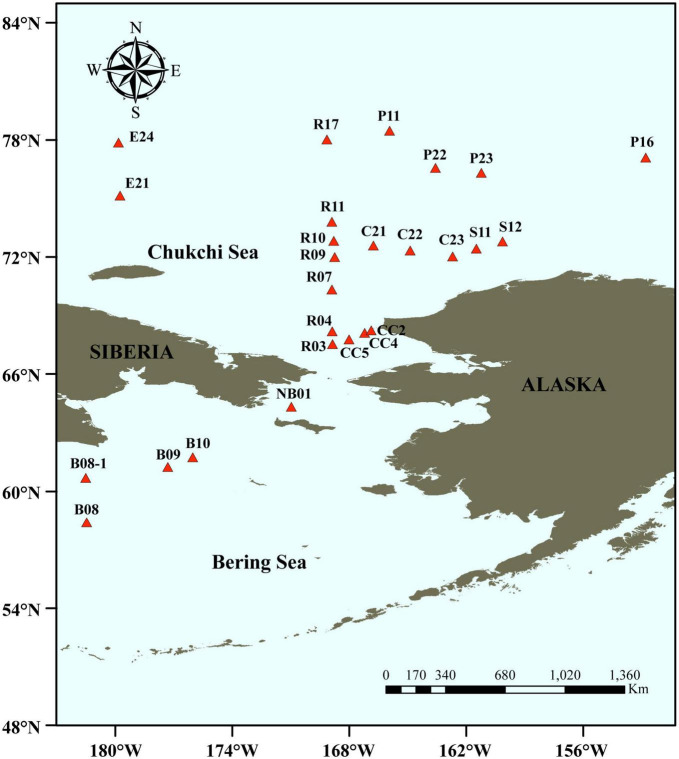
Sampling stations in the Bering-Chukchi shelf and adjacent sea areas. The names of the stations are described according to the 7th Chinese National Arctic Research Expedition.

Surface sediments in triplicates were collected from 26 stations in the Bering-Chukchi shelf and adjacent sea areas during the 7th Chinese National Arctic Research Expedition (CHINARE-7) from July to September, 2016, among which five stations locate in the Bering Sea (BS), thirteen stations in the Chukchi Sea (CS), five stations in the Chukchi Platform (CP), two stations in the Mendeleev Ridge (MR), and one station in the Canadian Basin (CB) (Figure 1). Location, water depth, and information of sediments of each station are listed in Table 1. General descriptions of the CHINARE-7 cruise in 2016 are presented in the cruise reports (Li and Xia, 2018). Sediments were collected using a box sampler, and the top 1 cm of sediments were sampled with a polyethylene spatula, and placed in a sealed plastic bag, and then stored in −80°C for further treatments.

**TABLE 1:** Information of sediment samples from the Bering-Chukchi shelf and adjacent sea areas.

Station	Sea area	Longitude	Latitude	Water depth (m)	Sediment character
B08	Bering Sea	178.538°E	58.40528°N	3,669	Yellowish-brown clayey silt
B08_1	Bering Sea	178.493°E	60.70361°N	169	Gray silt
B09	Bering Sea	177.304°W	61.25639°N	124	Gray silt
B10	Bering Sea	176.026°W	61.75861°N	97	Light yellow silty clay
NB01	Bering Sea	170.977°W	64.32583°N	40	Light gray medium coarse sand
C21	Chukchi Sea	166.766°W	72.60083°N	52	Cyan silty clay
C22	Chukchi Sea	164.877°W	72.33083°N	47	Bluish-yellow silty sand
C23	Chukchi Sea	162.708°W	72.02667°N	36	Gray silty sand
CC2	Chukchi Sea	167.221°W	68.11056°N	50	Yellowish-brown silty clay
CC4	Chukchi Sea	166.884°W	68.24306°N	36	Bluish-gray gravel silt
CC5	Chukchi Sea	168.013°W	67.78333°N	52	Light yellow gravel silt
R03	Chukchi Sea	168.868°W	67.53472°N	50	Cyan clay
R04	Chukchi Sea	168.879°W	68.20111°N	56	Gray fine sand
R07	Chukchi Sea	168.889°W	70.33944°N	39	Gray gravel sand
R09	Chukchi Sea	168.741°W	72°N	50	Light gray clayey silt
R10	Chukchi Sea	168.796°W	72.83556°N	61	Light gray clayey silt
S11	Chukchi Sea	161.486°W	72.43806°N	44	Gray clayey silt
S12	Chukchi Sea	160.151°W	72.79889°N	65	Light gray silty sand
P11	Chukchi Platform	165.932°W	78.48472°N	526	Yellowish-brown silty clay
P22	Chukchi Platform	163.588°W	76.56944°N	709	Yellowish-brown silty clay
P23	Chukchi Platform	161.228°W	76.32306°N	2,089	Brown silty clay
R11	Chukchi Platform	168.885°W	73.80194°N	155	Dark brown silty clay
R17	Chukchi Platform	169.143°W	78.02833°N	698	Yellowish-brown clayey silt
E21	Mendeleev Ridge	179.755°W	75.15417°N	550	Brown clayey silt
E24	Mendeleev Ridge	179.836°W	77.87639°N	1,575	Brown clayey silt
P16	Canadian Basin	152.804°W	77.10889°N	789	Yellowish-brown silty clay

### DNA extraction, PCR amplification, and sequencing

DNA in sediment samples was extracted by Qiagen’s PowerSoil DNA Isolation kit (Qiagen, Germany) following the manufacturer’s instructions. The 16S rRNA gene was amplified using the universal primers 515F (5′-GTGCCAGCMGCCGCGG-3′) (Liao et al., 2015) and 907R (5′-CCGTCAATTCMTTTRAGTTT-3′) (Yusoff et al., 2013) by an ABI GeneAmp^®^ 9700 PCR thermocycler (ABI, CA, USA). The primers work for both bacteria and archaea (Yusoff et al., 2013; Fu et al., 2018). PCR was performed using TransStart^®^ FastPfu DNA Polymerase (TransGen, China). The PCR reaction mixture included 4 μL 5 × Fast Pfu buffer, 2 μL 2.5 mM dNTPs, 0.8 μL each primer (5 μM), 0.4 μL Fast Pfu polymerase, 10 ng of template DNA, and ddH_2_O to a final volume of 20 μL. PCR amplification cycling conditions were as follows: initial denaturation at 95°C for 3 min, followed by 27 cycles of denaturing at 95°C for 30 s, annealing at 55°C for 30 s and extension at 72°C for 45 s, and single extension at 72°C for 10 min, and end at 4°C. All samples were amplified in triplicates. The PCR products were run on a 2% agarose gel and purified using the AxyPrep DNA Gel Extraction Kit (Axygen Biosciences, Union City, CA, USA) according to the manufacturer’s instructions and quantified using a Quantus Fluorometer (Promega, USA). Purified amplicons were pooled in equimolar amounts and paired-end sequenced on an Illumina MiSeq PE300 platform (San Diego, USA) according to the standard protocols by Majorbio Bio-Pharm Technology, Co., Ltd. (Shanghai, China).

### Bioinformatic analyses

The raw FASTQ files from the Illumina libraries were filtered by the fastp software (version 0.19.6)^1^ for quality control. The filtered sequences were merged by FLASH (version 1.2.7)^2^ with a minimum overlap of 10 bp and a maximum mismatch overlap ratio of 0.2. Then the optimized sequences were clustered into operational taxonomic units (OTUs) using UPARSE (version 7.1)^3^ with 97% sequence similarity level. The UCHIME software package was used to identify and remove probable chimeric sequences (version 8.0, Edgar et al., 2011). Non-microbiota (e.g., chloroplast and mitochondria) reads were removed via QIIME (version 2, Caporaso et al., 2010). The most abundant sequence for each OTU was selected as a representative sequence. The taxonomy of each OTU representative sequence was analyzed by RDP Classifier (version 2.2) against the SILVA 16S rRNA database (version 138,^4^ Quast et al., 2013) using confidence threshold of 0.7. The scientific names of prokaryotes were according to the descriptions by Oren and Garrity (2021), Oren and Göker (2023), and searched in BacDive (2023).^5^ The raw data were deposited into the National Center for Biotechnology Information (NCBI) Sequence Read Archive (SRA)^6^ with the accession number PRJNA979822.

### Analyses of biogenic elements

Sediments for biogenic elements analysis were dried in an oven at 40°C until reaching constant weight. The dried sediments were ground gently with an agate mortar and pestle, and sieved through a 100 μm-mesh, and then stored in a sealed glass vial. Total organic carbon (TOC) and total nitrogen (TN) was measured by a Perkin-Elmer 2400 Series II CHNS/O Analyzer (Perkin Elmer Inc., USA) after being treated with 10% HCl to remove carbonate in samples. Total phosphorus (TP) was measured by potassium persulfate digestion method (Thien and Myers, 1992). Biogenic silica (BSi) was measured by the molybdate blue spectrophotometric method after removing the carbonates and organics by 1 mol/L HCl and 10% H_2_O_2_, and digested using 0.5 mol/L Na_2_CO_3_ solution (Mortlock and Froelich, 1989). The quality assurance/quality control (QA/QC) was assessed by the analysis of blank reagents and five replicates of the certified reference material (Offshore Marine Sediment, GBW 07314). The precision of biogenic elements analysis was controlled to within 5%.

### Statistical analyses

To minimize the effects of sequencing depth on alpha and beta diversity measurement, DNA reads of each sample were normalized to the number of DNA reads in the sample with the fewest reads (76,898 reads in this study). The normalized data were used for further analysis. Alpha diversity indexes including OTU richness, Shannon, Simpson, Pielou evenness, and Goods coverage were calculated. Venn diagrams were calculated and plotted using the VennDiagram package (version 1.7.3, Lam et al., 2016). The Bray-Curtis (BC) index was used as a measure of similarity between samples. A distance matrix was computed with the BC index, and hierarchical cluster trees were constructed using the unweighted pair group method with arithmetic mean (UPGMA) with bootstrap support. Non-metric multidimensional scaling (NMDS) was constructed based on BC index using vegan (version 2.6-4, Dixon, 2003) and ggplot2 packages (version 3.4.4, Wickham, 2011), and permutation multivariate analysis of variance (PERMANOVA) was performed using the adonis function in the vegan package to test group differences (Guo et al., 2020). All these analyses were performed using the R (version 4.1.0).^7^

The redundancy analysis (RDA) was performed to reveal the impacts of biogenic elements in explaining the distribution patterns of prokaryotic communities in 26 surface sediments using Canoco5.02 (Ter Braak and Šmilauer, 2012). All data were logarithmically transformed to obtain the equal weight of the elements in RDA analysis. Spearman correlation coefficients were used to judge the relationships between relative DNA reads of prokaryotes and biogenic elements by SPSS 25.0 (De Winter et al., 2016). The histogram and pie figures were drawn with Microsoft Office Excel 2019.

Co-occurrence networks were constructed by using the “Hmisc” package based on Spearman correlation (version 5.0-1, Harrell and Harrell, 2019). Only OTUs with relative abundance > 0.001% and appeared in three or more samples were used in the analysis. Visualization of the co-occurrence network was performed by using Gephi (Gephi 0.1.0 beta). Keystone taxa are defined as those having a high degree, high closeness centrality, and low betweenness centrality values in the co-occurrence networks (Berry and Widder, 2014; Banerjee et al., 2018). For the network at class level, classes with ≥ 9 degrees, closeness centrality > 0.4, and betweenness centrality < 10 were chosen as putative keystone classes. For OTU-OTU networks, OTUs with closeness centrality > 0.2, betweenness centrality < 10, and degrees ≥ 10, 6, and 4 were chosen as putative keystone OTUs for samples from the whole zone, the Bering-Chukchi shelf, and the northern deep seas, respectively.

## Results

### Community structure of prokaryotes

**FIGURE 2:**
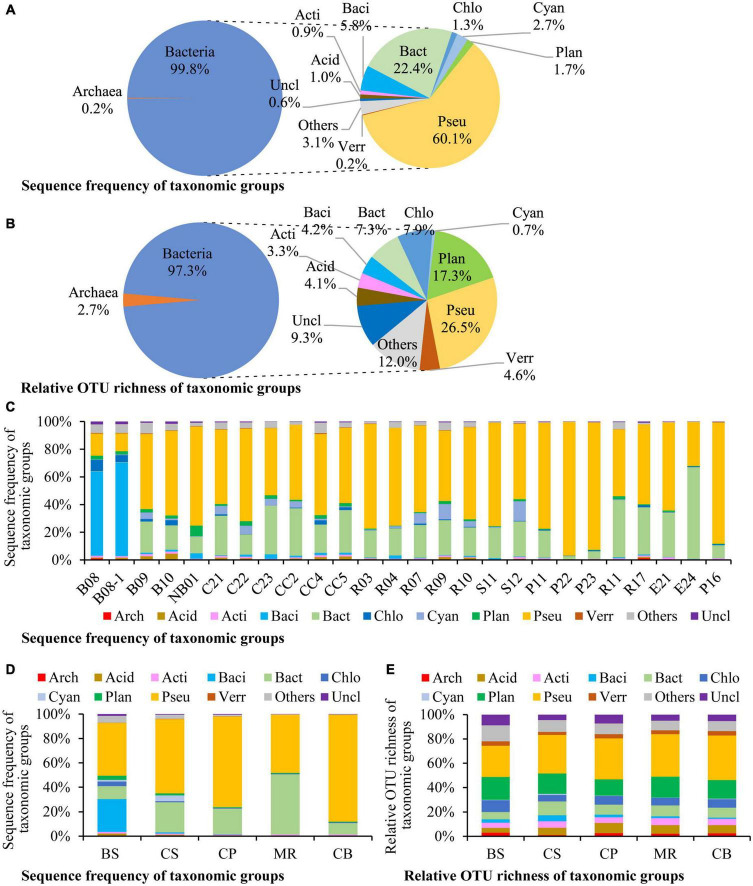
Sequence frequency **(A,C,D)** and relative OTU richness **(B,E)** of taxonomic groups in the overall samples **(A,B)**, each station **(C)**, and each sea area **(D,E)**. The small circles in the right side of panels **(A,B)** show relative abundances of each division to bacterial reads/OTUs. Bacterial divisions are represented by their first four characters. Arch: Archaea, Acid: Acidobacteriota, Acti: Actinomycetota, Baci: Bacillota, Bact: Bacteroidota, Chlo: Chloroflexota, Cyan: Cyanobacteria, Plan: Planctomycetota, Pseu: Pseudomonadota, Verr: Verrucomicrobiota, Uncl: those unclassified to the division level, Others: All of the other bacterial divisions. BS, the Bering Sea; CS, the Chukchi Sea; CP, the Chukchi Platform; MR, the Mendeleev Ridge; CB, the Canadian Basin.

Altogether 106 species, 411 genera in 72 classes of 45 phyla were detected in this study (Supplementary Table 1). There were 114 OTUs (2.2%) identified at the species level, and 1,067 OTUs (20.6%) at the genus level. Most of the microbes identified to the species level belong to the aerobic marine taxa, and also include some anaerobic, parasitic, and phototrophic taxa (Supplementary Table 2). Bacteria dominated in prokaryotic community, which contributed to 99.8% of the prokaryotic DNA reads and 97.3% of the OTU richness (Figures 2A, B). Pseudomonadota was the most dominant phylum, accounting for 60.1% of the overall prokaryotic reads, followed by Bacteroidota (22.4%, Figure 2A). Pseudomonadota dominated in most stations, Bacteroidota dominated at station E24, and Bacillota dominated at stations B08 and B08-1 (Figure 2C). Prokaryotic community structure was different among sea areas (Figure 2D). Pseudomonadota and Bacillota co-dominated in the BS. Pseudomonadota was predominated in the CS, CP, and CB, and its proportions increased from the southern CS to the northern CB gradually. Prokaryotic community in the MR was co-dominated by Bacteroidota and Pseudomonadota. Phototrophic cyanobacteria occurred in most samples, and in high abundances in the CS (Figure 2C).

**FIGURE 3:**
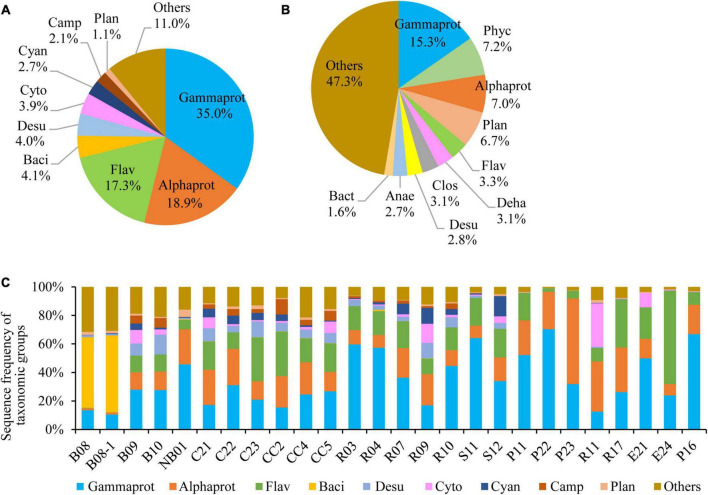
Bacterial community structure at class level. **(A)** Sequence frequency of taxonomic groups, **(B)** Relative OTU richness of taxonomic groups, **(C)** Sequence frequency of taxonomic groups in each station. Gammaprot: Gammaproteobacteria, Phyc: Phycisphaerae, Alphaprot: Alphaproteobacteria, Flav: Flavobacteriia, Deha: Dehalococcoidia, Clos: Clostridia, Baci: Bacilli, Desu: Desulfobacterota, Cyto: Cytophagia, Cyan: Cyanobacteria, Camp: Campylobacterota, Plan: Planctomycetacia, Anae: Anaerolineae, Bact: Bacteroidia, Others: including all other classes.

Pseudomonadota was the most diverse phylum in this study (Figure 2B), with a total of 1,373 OTUs, followed by Planctomycetota (897 OTUs), Chloroflexota (410 OTUs), Bacteroidota (381 OTUs), Verrucomicrobiota (237 OTUs), Bacillota (216 OTUs), Acidobacteriota (214 OTUs), and Actinomycetota (173 OTUs). The profiles of OTU richness were similar in the five sea areas, with abundant richness of Pseudomonadota and Planctomycetota (Figure 2E). Gammaproteobacteria was the first dominant bacterial class, contributing 35.0% of the bacterial reads (Figure 3A), and it was also the most diverse class (Figure 3B). The bacterial community varied among samples, and Gammaproteobacteria was the first dominant group in 16 samples, with relative reads of 10.4–70.3% to the bacterial reads (Figure 3C). Alphaproteobacteria dominated in four samples, with relative reads of 1.4–60.2%. Flavobacteriia also dominated in four samples, with relative reads from 0.2 to 65.7%. Bacilli was dominant at B08 and B08-1 with relative reads ca. 50%, however, less than 0.5% in other samples.

### Abundant and rare OTUs in the prokaryotic community

**FIGURE 4:**
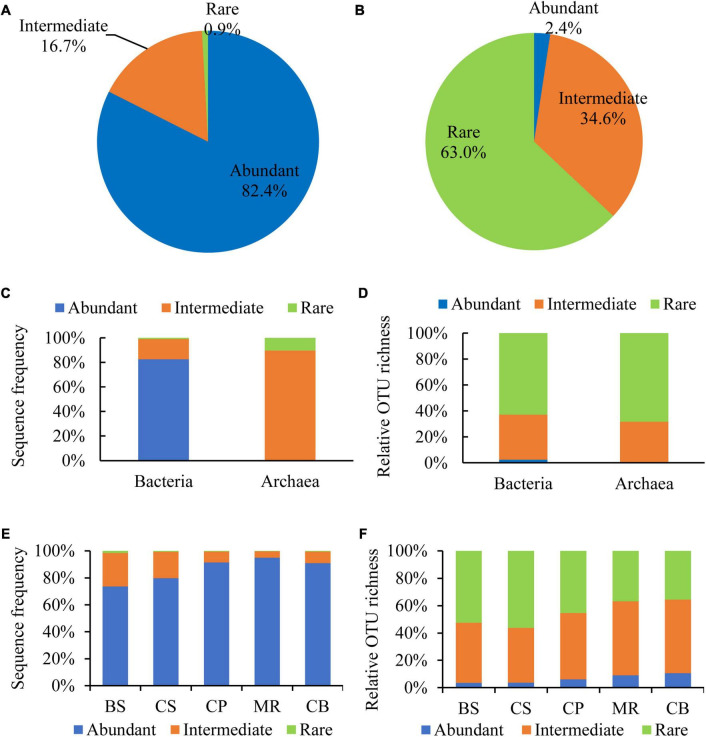
Sequence frequency **(A,C,E)** and relative OTU richness **(B,D,F)** of abundant (≥1% in one sample), intermediate (intermediately abundant, 0.01–1%), and rare (<0.01% in all samples) OTUs in the overall samples **(A,B)**, bacteria **(C)** and archaea **(D)**, and sea areas **(E,F)**.

Abundant and rare OTUs were defined according to the definition by Pedrós-Alió (2006) and Galand et al. (2009) with a representation ≥ 1% within a sample and < 0.01% within all samples. Intermediately abundant OTUs were referred to those with a relative abundance between 0.01 and 1%. Abundant, intermediately abundant, and rare OTUs contributed 82.4, 16.7, and 0.9% of the DNA reads, and 2.4, 34.6, and 63.0% of the OTU richness, respectively (Figures 4A, B). All of the abundant OTUs belonged to bacteria with the relative reads of 82.6% to the bacterial reads (Figure 4C). While the intermediately abundant OTUs dominated in the archaeal reads with the relative reads of 89.8%. Rare OTUs occupied most of OTU richness of both bacteria and archaea, accounting for 62.9 and 68.3%, respectively, followed by intermediately abundant OTUs, accounting for 34.7 and 31.7%, respectively (Figure 4D). Proportions of DNA reads of abundant OTUs increased from the BS to the CB, from < 80% in the BS and the CS to > 90% in the three northern deep seas (Figure 4E). The relative richness of abundant OTUs also showed a general increase trend from the southern BS sea area to the northern sea areas (Figure 4F).

### Alpha and beta diversity indexes

**FIGURE 5:**
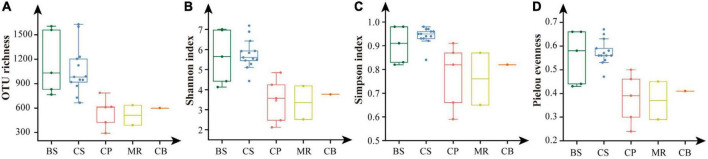
Alpha diversity indexes of prokaryotes in the five sea areas of the Bering-Chukchi shelf and adjacent sea areas. **(A)** OTU richness, **(B)** Shannon, **(C)** Simpson, **(D)** Pielou evenness. BS, the Bering Sea; CS, the Chukchi Sea; CP, the Chukchi Platform; MR, the Mendeleev Ridge; CB, the Canadian Basin.

Shannon, Simpson, and Pielou evenness ranged from 2.12 to 7.19, from 0.59 to 0.98, and from 0.24 to 0.67, respectively (Table 2). Alpha diversity indexes were significantly higher in the BS and CS, with OTU richness > 1,000 OTUs, Shannon > 5.6, Simpson > 0.9, Pielou evenness > 0.55 (Figure 5). Values of alpha diversity indexes were comparable in the three sea areas of the northern deep seas, with the averages of 513–601 OTUs, 3.35–3.77 for Shannon, 0.76–0.82 for Simpson, and 0.37–0.41 for Pielou evenness, respectively (Table 2).

**TABLE 2:** Alpha diversity indexes of prokaryotes in surface sediments from the Bering-Chukchi shelf and adjacent sea areas.

Station	Richness	Shannon	Simpson	Pielou evenness	Goods coverage
B08	1,033	4.42	0.83	0.44	0.9984
B08_1	767	4.13	0.82	0.43	0.9987
B09	1,558	6.97	0.98	0.66	0.9941
B10	1,606	7.01	0.98	0.66	0.9949
NB01	831	5.65	0.91	0.58	0.9987
**BS Ave**	**1,159**	**5.64**	**0.90**	**0.55**	**0.9970**
C21	1,229	5.84	0.94	0.57	0.9947
C22	1,124	6.43	0.97	0.63	0.9961
C23	666	5.11	0.93	0.54	0.9974
CC2	949	5.28	0.92	0.53	0.9959
CC4	1,629	7.19	0.98	0.67	0.9937
CC5	1,599	6.89	0.97	0.65	0.994
R03	874	5.45	0.93	0.56	0.9966
R04	946	5.55	0.95	0.56	0.996
R07	983	5.61	0.96	0.56	0.9958
R09	1,205	5.93	0.95	0.58	0.9949
R10	988	5.92	0.96	0.59	0.9958
S11	729	4.43	0.84	0.47	0.9967
S12	920	5.5	0.94	0.56	0.996
**CS Ave**	**1,065**	**5.78**	**0.94**	**0.57**	**0.9957**
P11	611	3.57	0.82	0.39	0.9978
P22	292	2.47	0.66	0.3	0.9988
P23	424	2.12	0.59	0.24	0.9986
R11	787	4.85	0.91	0.5	0.9966
R17	615	4.24	0.87	0.46	0.9989
**CP Ave**	**546**	**3.45**	**0.77**	**0.38**	**0.9981**
E21	636	4.18	0.87	0.45	0.9973
E24	390	2.52	0.65	0.29	0.9983
**MR Ave**	**513**	**3.35**	**0.76**	**0.37**	**0.9978**
P16	601	3.77	0.82	0.41	0.998

BS, the Bering Sea; CS, the Chukchi Sea; CP, the Chukchi Platform; MR, the Mendeleev Ridge; CB, the Canadian Basin. Bold font indicates the average value of the sea area.

**FIGURE 6:**
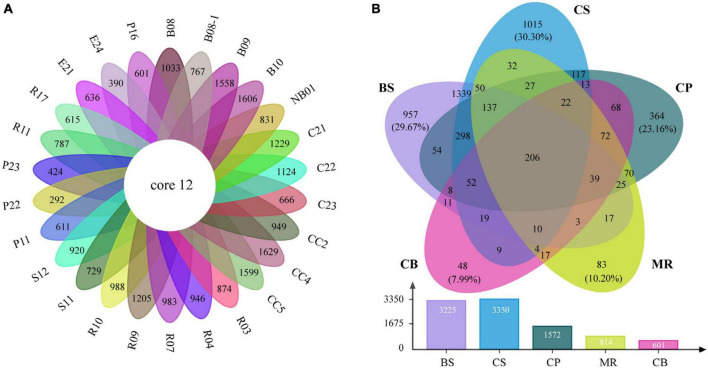
Venn diagrams highlighting the degree of overlap of prokaryotic OTUs among samples **(A)** and the five sea areas **(B)**. BS, the Bering Sea; CS, the Chukchi Sea; CP, the Chukchi Platform; MR, the Mendeleev Ridge; CB, the Canadian Basin.

The venn diagrams highlighted the differences of prokaryotic communities among samples and sea areas (Figure 6). Only 12 OTUs (0.2%) were shared among the 26 samples including 7 abundant OTUs and 5 intermediately abundant OTUs (Supplementary Table 3). OTU richness in each sea area ranged between 601 and 3,350 OTUs (Figure 6B). OTU richness was higher in sea areas with more stations such as the CS, and decreased in the northern deep seas. For example, 3,225 OTUs were recorded in five samples from the BS, while only 1,572 OTUs in five samples from the CP. There were 206 OTUs (4.0%) shared among the five sea areas, including 53 abundant OTUs, 142 intermediately abundant OTUs, and 11 rare OTUs (Supplementary Table 4). A large number of unique OTUs were recorded in the CS, BS, and CP, which were 1,015 OTUs (30.3%), 957 OTUs (29.7%), and 364 OTUs (23.2%), respectively. The numbers of unique OTUs decreased to 83 OTUs (10.2%) in the MR and 48 OTUs (8.0%) in the CB. Only two OTUs of the unique OTUs belonged to the abundant OTUs, while rare OTUs accounted for 75.7–95.8% of the unique OTUs in each sea area, and increased from the BS and CS to the northern deep seas.

**FIGURE 7:**
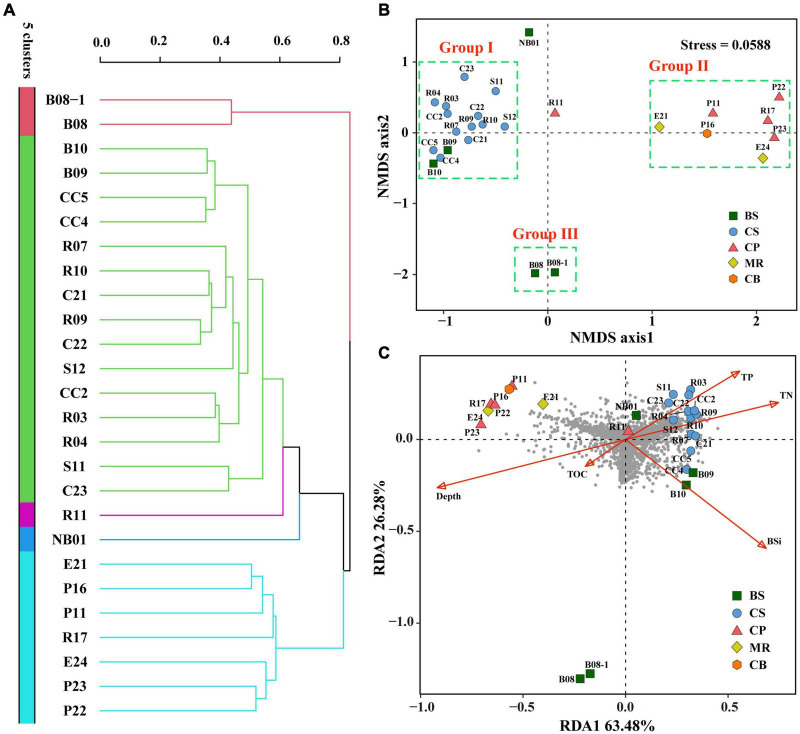
Cluster, Non-metric multidimensional scaling (NMDS), and Redundancy analysis (RDA) of the twenty-six sediment samples from the Bering-Chukchi shelf and adjacent sea areas based on prokaryotic community. Cluster and NMDS analyses were constructed based on the Bray-Curtis (BC) index. RDA was conducted based on environmental parameters and prokaryotic community. **(A)** Cluster analysis. **(B)** NMDS analysis. **(C)** RDA plot ordination sampling stations, prokaryotic OTUs (dark-gray circles), and biogenic elements and water depth (red arrows). BS, the Bering Sea; CS, the Chukchi Sea; CP, the Chukchi Platform; MR, the Mendeleev Ridge; CB, the Canadian Basin.

Cluster analysis grouped the 26 samples into five groups (Figure 7A), including a small group of B08 and B08-1, a large group of all samples from the CS together with B09 and B10 (green group). Station R11 in the CP and NB01 in the BS were ungrouped, and then clustered to the green group. While the other four samples from the CP and all samples from the MR and CB were clustered together to a blue-green group. The result of NMDS were comparable (Figure 7B), B09 and B10 together with all samples from the CS clustered together at the negative axis of the NMDS axis 1, while all samples from the northern deep seas clustered to the positive axis of the NMDS axis 1. Samples from B08 and B08-1 and samples from NB01 and R11 situated at the negative and positive axis of NMDS axis 2, respectively. Based on the clustering and NMDS analysis results, the 26 stations can be divided into five groups. Group I included all samples from the CS together with B08 and B09. Group II included all samples from the MR, the CB, and the CP stations except for R11, Group III included B08 and B08-1, and the other two stations were ungrouped (Figure 7B). Significant differences were found between the three groups based on results of PERMANOVA (*r*^2^ = 0.264∼0.437, *p* < 0.05 or < 0.01) (Table 3). The results suggested that the prokaryotic communities were similar in samples from the Bering-Chukchi shelf (south of 73°N with water depth less than 150 m), and were quite different in samples from the northern deep seas (north of 75°N with water depth greater than 500 m).

**TABLE 3:** Permutational multivariate analysis of variance (PERMANOVA) to evaluate the distances between groups in the Non-metric multidimensional scaling (NMDS).

Pairs	Df	Sums of Sqs	F. Model	*R* ^2^	*p*-value	p. adjusted
I vs. II	1	1.637	7.159	0.264	0.001	0.009**
I vs. III	1	1.274	6.019	0.286	0.003	0.014*
II vs. III	1	1.101	5.438	0.437	0.028	0.037*

*p < 0.05,

**p < 0.01.

### Correlation and redundancy analyses between prokaryotic community and environmental factors

Bacterial OTU richness and diversity indexes of prokaryotes were negatively correlated with water depth (*r* = −0.499∼−0.669, *p* < 0.01), while archaeal OTU richness was positively correlated with water depth (*r* = 0.353, *p* = 0.077, Table 4). Latitude showed negative correlations with all alpha diversity indexes for both bacteria and archaea. There were significant positive correlations between the bacterial OTU richness and all diversity indexes of prokaryotes (*r* = 0.890∼0.932, *p* < 0.01), indicating that bacteria are important contributors to prokaryotic biodiversity. The bacterial OTU richness and all alpha diversity indexes showed insignificantly negative correlations with TOC (*r* = −0.153 ∼−0.256, *p* > 0.05), however, showed significantly or insignificantly positive correlations with other biogenic elements.

**TABLE 4:** Spearman correlation coefficients between alpha diversity indexes and environmental factors.

			OTU richness								
			**Archaea**	**Bacteria**	**Prokaryotes**	**Depth**	**Latitude**	**TOC**	**BSi**	**TN**	**TP**
OTU richness	Archaea	r	1	0.249	0.266	0.353	-0.228	0.247	0.023	-0.245	-0.523**
		*p*		0.220	0.189	0.077	0.263	0.223	0.913	0.228	0.006
	Bacteria	r	0.249	1	0.999**	-0.509**	-0.655**	-0.153	0.560**	0.629**	0.372
		*p*	0.220		0.000	0.008	0.000	0.456	0.003	0.001	0.062
	Prokaryotes	r	0.266	0.999**	1	-0.499**	-0.667**	-0.157	0.567**	0.614**	0.360
		*p*	0.189	0.000		0.010	0.000	0.444	0.003	0.001	0.071
	Shannon	r	0.070	0.932**	0.928**	-0.658**	-0.564**	-0.239	0.397*	0.586**	0.418*
		*p*	0.735	0.000	0.000	0.000	0.003	0.240	0.045	0.002	0.034
	Simpson	r	0.039	0.890**	0.884**	-0.630**	-0.489*	-0.241	0.388	0.605**	0.464*
		*p*	0.852	0.000	0.000	0.001	0.011	0.235	0.050	0.001	0.017
	Pielou evenness	r	0.016	0.895**	0.889**	-0.669**	-0.543**	-0.256	0.370	0.561**	0.433*
		*p*	0.938	0.000	0.000	0.000	0.004	0.207	0.063	0.003	0.027

*p < 0.05,

**p < 0.01.

Redundancy analysis was conducted based on prokaryotic community, biogenic elements, and water depth (Figure 7C). RDA 1 and RDA 2 explained 63.5 and 26.3% of the environmental and biological variables, respectively. TN, TP, and BSi had high positive contributions to axis RDA 1, while water depth represented a high negative contribution to RDA 1. TN and TP showed low to moderate positive contributions to axis RDA2, and other environmental variables had negative contributions to RDA2. Most of the prokaryotic OTUs (dark gray circles) scattered around the origin of coordinates and the positive direction of RDA1, indicating that the distribution of these OTUs was affected by TN, TP, and BSi. While some OTUs distributed in the third quadrant, indicating that they were influenced by water depth and TOC, mostly by water depth. However, some other OTUs scattered in the second quadrant, suggesting that their distribution was not significant influenced by any of above environmental parameters. All samples from the CS together with R11 in the CP and three samples from the BS (B09, B10, and NB01) closely distributed at the positive direction of RDA1, indicating high TN, TP, BSi, and OTU richness in these samples. Seven samples from the northern deep seas were distributed in the second quadrant, which reflected high water depth and relatively low OTU richness at these stations. Furthermore, B08 and B08-1 were far away from other stations, suggesting distinct prokaryotic community and environmental characteristics in these two stations.

### Potential interactions among prokaryotic communities

**FIGURE 8:**
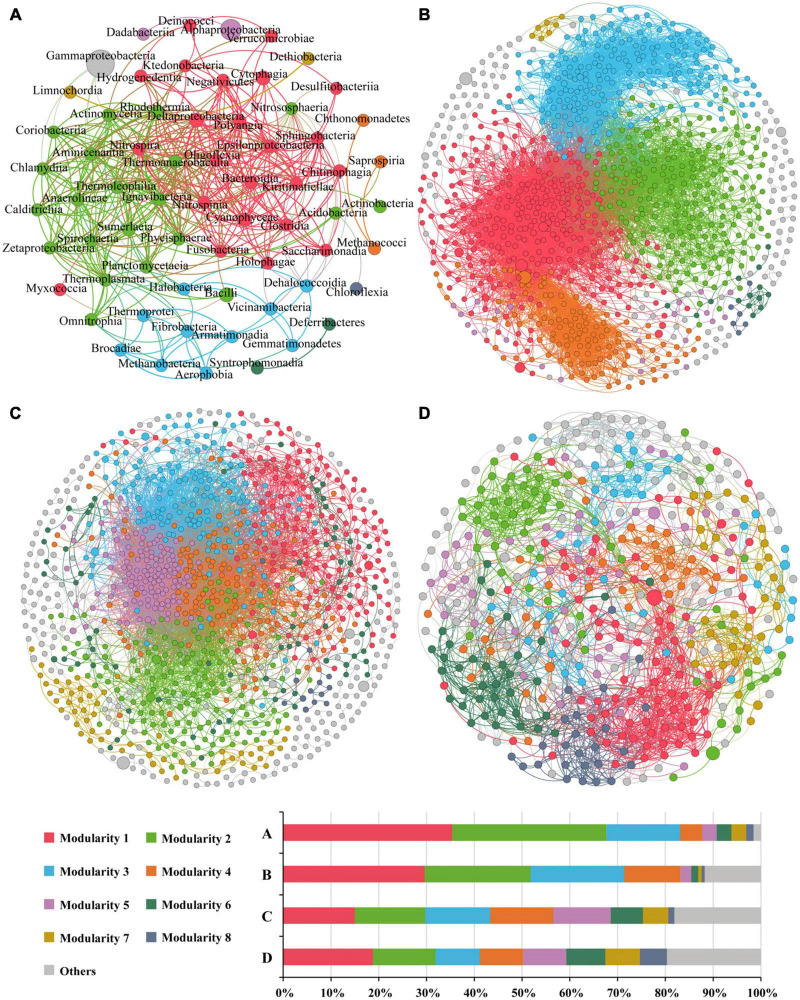
The co-occurrence networks of prokaryotic communities at the class level **(A)**, and OTU-OTU interactions in the whole zone **(B)**, the Bering-Chukchi shelf [Group I in the NMDS, **(C)**] and the northern deep seas [Group II in the NMDS, **(D)**]. Only statistically significant correlations between different groups (Spearman correlation ≥ |0.5| and *p* < 0.05 for the prokaryotes at class level, and Spearman correlation ≥ |0.8| and *p* < 0.01 for OTU-OTU) are shown. The nodes represent unique sequences in the datasets. Node sizes correspond to the relative abundance of DNA reads. The nodes colored by modularity class, colored nodes (except light gray) represent the major groups and light gray represents all modules except the major modules. The thickness of the edges represents the correlation between nodes.

Overall, there were 405 links in the network based on class level (Figure 8A), with positive correlation accounting for 91.1%. Altogether there were nine modularity classes, and the top two modules accounted for 35.4 and 32.3% of the nodes, respectively. However, the top abundant classes (reflected by the node size) were not in the major modules. Gammaproteobacteria formed a module of its own, and only negatively correlative to five classes. Alphaproteobacteria had only one link, and formed a module with only one other class. Eight classes were considered as keystone taxa, all of which belonged to the first two modules (Supplementary Table 5). Modules 1 and 2 showed significant or insignificant negative correlation with water depth (*r* = −0.538, *p* = 0.005 and *r* = −0.303, *p* = 0.132, respectively) (Supplementary Table 6), suggesting that these two modules (contributing to 67.7% of the nodes) might represent microbial communities in the shallow Bering-Chukchi shelf. The significantly positive correlations of the two modules with BSi (*r* = 0.459, *p* = 0.010 and *r* = 0.570, *p* = 0.002, respectively) reflected influences of high diatom productivity on the distribution of the modules. Module 3 was positively correlated with water depth (*r* = 0.696, *p* = 0.000), suggesting that this module might represent the microbial communities in the northern deep seas. Most of the keystone classes showed significantly or insignificantly negative correlation with water depth, however, mostly positive correlation with BSi, TN, and TP (Supplementary Table 7).

The OTU-OTU association network for the whole zone were depicted in Figure 8B, which were consisted of 975 nodes and 12,460 edges (positive edges accounted for 96.7%). The high values of modularity (MD, 0.629), average degree (AD, 25.56), average clustering coefficient (ACC, 0.530) (Table 5) suggested good modular structure and high network complexity (Zhang et al., 2022). Eleven keystone OTUs were found, and all belonged to the intermediately abundant OTUs (Supplementary Table 8). Positive correlations were among the top six major modules except for module 4, and these five modules demonstrated similar correlations with environmental factors, i.e., negative correlations with depth and TOC, positive correlations with BSi, TN, and TP (Supplementary Table 9). On the other hand, the keystone OTUs did not show clear correlations with environmental factors (Supplementary Table 10).

**TABLE 5:** Comparison of the topological properties of the co-occurrence networks of prokaryotic communities in the whole zone and samples from the Bering-Chukchi shelf (Group I) and the northern deep seas (Group II).

	Nodes	Edges	AD	ND	GD	MD	CC	ACC	APL	Number of modules
Whole zone at Class level	65	405	12.46	7	0.195	0.383	4	0.621	2.30	9
Whole zone	975	12,460	25.56	15	0.026	0.629	36	0.530	4.01	53
Group I	1,015	10,716	21.12	16	0.021	0.367	41	0.406	4.42	100
Group II	442	2,285	10.34	13	0.023	0.961	1	0.479	4.69	31

AD, average degree; ND, network diameter; GD, graph density; MD, modularity; CC, connected components; ACC, average clustering coefficient; APL, average path length.

The OTU-OTU association network for samples from the Bering-Chukchi shelf (Group I in the NMDS) were illustrated in Figure 8C, including 1,015 nodes and 10,716 edges (positive edges accounted for 97.7%). Some of the dominant OTUs (reflected by the node size) were outside the colored major modules but in the gray edge modules. The networks exhibited the low values of MD (0.367) and ACC (0.406) (Table 5), suggesting weak modular structure in Group I. While high AD value (21.12) reflected high connections and close co-occurrences among OTUs. Ten keystone OTUs were found, and most of them belonged to the intermediately abundant OTUs (Supplementary Table 11). Most of the major modules were positively correlated, however, the relationships between modules and keystone OTUs and environmental factors were unclear (Supplementary Tables 12, 13).

The topological properties of the co-occurrence network for samples from the northern deep seas (Group II in the NMDS) were charactered by high MD (0.961), average path length (APL, 4.69), and low edges (2,285), AD (10.34), and module number (31) (Table 5). The results suggested strong modularity and differences among samples (Figure 8D). Only four keystone OTUs were obtained (Supplementary Table 14). The relationships between modules and environmental factors were unclear (Supplementary Table 15). Two keystone OTUs (OTU4099 and OTU3892) had a correlation coefficient of 1.000 (Supplementary Table 16), suggesting the same distribution pattern of them.

## Discussion

Water depth and latitude influence the prokaryotic assemblages significantly in this study. Both cluster and RDA analyses showed that samples from the northern deep seas are grouped together, while the shallow northern BS and CS stations are clustered as a large group. In addition, alpha diversity indexes were significantly higher in the BS and CS than those in the northern deep seas. The results suggested different prokaryotic community assemblages between the Bering-Chukchi shelf and the northern deep seas. The Bering-Chukchi shelf is well known to be one of the most productive regions in the world (Huntington et al., 2020; Lalande et al., 2021), which can produce large amounts of phytodetritus to the sea seafloor specially during the summer bloom seasons, and provide sufficient nutrients for the growth of bacteria. However, the large ice coverage and deep water in the northern deep seas hinders and decrease organic matters and nutrients sinking to the sea bottom, and thus result in a decline in benthic microbial diversity.

The abundant taxa are thought to be the most active and important in fluxes of dissolved organic matter (Cottrell and Kirchman, 2003). However, abundant species represent only a small portion of microbial diversity (Galand et al., 2009). Abundant OTUs (≥1% in one sample) contributed most of the DNA reads, while the intermediately abundant and rare OTUs shared most of OTU richness in our study. Furthermore, the relative reads of abundant OTUs increased from the south to the north, suggesting that the abundant taxa may play more important ecological functions in the northern deep seas. Meanwhile, the extreme cold-dark environment and deep-water may also increase the abundance of certain specific resistant sedimentary bacteria in the northern deep seas. There were no rare OTUs in the 12 core OTUs shared among all 26 samples, and only 11 rare OTUs in the 206 OTUs shared among the five sea areas. However, most of the unique OTUs in each sample and sea areas were rare OTUs. Our results suggested the rare OTUs did not have a cosmopolitan distribution, which is agreed with Galand et al. (2009) in discussing the ecology of the rare microbial biosphere of the Arctic Ocean.

The co-occurrence network diagrams showed complex relationships among microbial taxa, reflected by high links, degrees, and strong inter-connections between modules, which suggested a stable microbial community structure in the study area (Röttjers and Faust, 2018). Bacteria co-occur because of positive interactions such as cooperative relationships or similar niche preferences, and co-exclude each other because of negative interactions such as competition or antagonism (Freilich et al., 2018; Li et al., 2019; Zhang et al., 2022). The majority of positive interactions (81.8–97.7%) in this study indicated that there are primarily symbiotic and/or cooperative relationships in the microbial network (Li et al., 2019; Zhang et al., 2022). Generally, taxa in the same module have similar niches, and they co-occurred together (Faust and Raes, 2012). One hundred modules were derived from the network of Group I (the Bering-Chukchi shelf), which may be caused by the rich environmental habitats for microbiomes in this high productivity area. However, graph density and modularity were the lowest in Group I within all co-occurrence networks, suggesting low variations among the whole samples. On the other hand, the network of Group II (the northern deep seas) showed very high modularity (0.961), which may be related to the diverse environment of the sea areas. Most of the dominant taxa (class or OTU) were located at the edge of the networks with few links with other taxa and week interaction with the major modules. The strong co-exclusion pattern of the dominant taxa suggested that they might have some competitive and/or antagonistic effects with other taxa (Freilich et al., 2018; Zhang et al., 2022).

Keystone species are commonly defined as species that exert a disproportionately large effect on the ecosystem relative to their abundance (Power et al., 1996). Most keystone OTUs were intermediately abundant OTUs with the relative abundance between 0.01 and 1% in this study, suggesting that taxa with moderate biomass might have considerable impacts on the structure and function of the microbial community (Banerjee et al., 2018). Keystone OTUs belonging to the phylum Chloroflexota were recorded in samples from the whole zone, Group I, and Group II. Chloroflexota is known to perform both anoxygenic photosynthesis and a unique C fixation metabolic process, the 3-hydroxypropionate (3HP) bicycle (Shih et al., 2017). The key roles of Chloroflexota may be probably related to their anaerobic photosynthesis in the benthic environment. The class Gammaproteobacteria was the most dominant class in this study, and keystone OTUs belonging to Gammaproteobacteria were recorded in samples from the whole zone and Group I. Given their global distribution and high abundance, Gammaproteobacteria may drive important parts of marine carbon and sulfur cycles (Dyksma et al., 2016). Keystone OTUs belonging to the phylum Bacteroidota also shared in samples from the whole zone and Group I, which are regarded as copiotrophic and fast growth taxa and can rapidly grow to high abundances during and after phytoplankton blooms (Brüwer et al., 2023).

It might be more informative by using the amplicon sequence variants (ASVs) to denoise the data and subsequently cluster these ASVs into distance OTUs. Since we only compared between our own samples, biases are similar across samples. Therefore, using 97% similarity cutoff to define OTUs in this study is acceptable. Besides, the lack of coverage at the species level for many taxa may hinder the correct interpretation of metabarcoding data. In this study, only 114 OTUs (2.2%) and 1,067 OTUs (20.6%) were identified at the species and genus level, respectively. We find it difficult to discuss the exact ecological characteristic of some specific OTUs, such as the core OTUs shared among stations and sea areas, the unique OTUs in each sea area, and the keystone OTUs. According to the ecological characteristic of 106 species identified (Supplementary Table 2), most of them belong to aerobic marine species. Walker et al. (2023) has reported that the anaerobic taxa generally increased with sediment depth in sediments from the northern Bering and southern Chukchi Sea shelves. Therefore, the dominance of aerobic microbiota in the upper 1 cm sediment in our study is reasonable. However, a variety of anaerobic species in class Clostridia was present in the CS (Supplementary Table 2), which may be caused by the local hypoxia of benthic environment after the summer algal blooms. Generally, nutrients in sediments influenced the benthic bacterial community composition (Dong et al., 2017). The correlation analysis showed that water depth and latitude had significant effects on the community structure of bacteria and prokaryotes, and OTU richness and diversity indexes mostly showed positive correlations with TN, TP, and BSi (Table 4). The results suggest that water depth, latitude, and nutrients play important roles in the community structure and distribution of prokaryotes in surface sediments from the Bering-Chukchi shelf and adjacent sea areas.

## Conclusion

This study enriched our understanding of prokaryotic community in surface sediments from the Bering-Chukchi shelf and adjacent sea areas. Bacteria dominated in prokaryotic community. However, archaeal DNA reads and OTU richness gradually increased in deeper samples. The prokaryotic community was dominated by Gammaproteobacteria, Alphaproteobacteria, and Flavobacteriia. The phototrophic Cyanobacteria occurred widely with the highest relative reads of 14.2%. Prokaryotic community assemblages were quite different in the northern deep seas compared to the Bering-Chukchi shelf, represented by the lowered diversity and the increased abundant OTUs. The bacterial community structure was significantly different at the two southernmost stations of the study, which was dominated by Bacillota rather than Pseudomonadota and/or Bacteroidota at other stations. Correlation analysis showed that latitude, water depth, and nutrients were important factors affecting the prokaryote community structure. Abundant OTUs distributed widely in the study area. Most of the keystone OTUs were intermediately abundant OTUs, suggesting that taxa with moderate biomass might have considerable impacts on the structure and function of the microbial community. The strong co-exclusion pattern of the dominant taxa suggested that they might have some competitive and/or antagonistic effects with other taxa.

## Data availability statement

The datasets presented in this study can be found in online repositories. The names of the repository/repositories and accession number(s) can be found below: https://www.ncbi.nlm.nih.gov/, PRJNA 979822.

## Author contributions

YT: Software, Writing—original draft, Writing—review and editing. CX: Data curation, Software, Writing—review and editing. HO: Investigation, Writing—review and editing. MW: Methodology, Writing—review and editing. HZ: Data curation, Writing—review and editing. JG: Investigation, Writing—review and editing. LX: Conceptualization, Supervision, Validation, Writing—review and editing. ZW: Data curation, Supervision, Visualization, Writing—original draft.

## Footnotes

1
https://github.com/OpenGene/fastp

2
http://www.cbcb.umd.edu/software/flash

3
http://drive5.com/uparse/

4
https://www.arb-silva.de/

5
https://bacdive.dsmz.de/strain/14279

6
https://www.ncbi.nlm.nih.gov/sra/

7
https://www.R-project.org/
